# Supplementary material for: Impact of introducing fluorescent microscopy on hospital tuberculosis control: A before-after study at a high caseload medical center in Taiwan
Source: PLoS One. 2020 Apr 3;15(4):e0230067. doi: 10.1371/journal.pone.0230067 (PMC7122812; doi:10.1371/journal.pone.0230067)
Supplement: S1 Table — (DOCX) [file pone.0230067.s001.docx]

**S1 Table. Tuberculosis control practices, 2001–2014**

| Interventions | Items | Implementations |
| --- | --- | --- |
| Baseline | Every ward was equipped with one respiratory isolation room (48 isolation rooms in total) | Before 2001 |
|  | Physicians were educated and encouraged to put the patients in respiratory isolation once infectious pulmonary TB was suspected based on their clinical presentations (prolonged cough for more than 3 weeks) or images (upper-lobe or cavity lesions). | Before 2001 |
|  | It is mandatory to isolate patients with positive AFS sputum | Before 2001 |
|  | The fluorometric BACTEC technique (BACTEC Mycobacterium Growth Indicator Tube [MGIT] 960 system, Becton-Dickinson, Sparks, MD) was implemented. | After July 1998 |
| Enhanced contact investigation | Annual chest radiography examination for high-risk healthcare workers | Since 2002 |
|  | Establishment of TB case managers to facilitate contact investigation, education and follow-up for all healthcare workers who exposed to hospitalized patients with pulmonary tuberculosis after delayed respiratory isolation | Since 2004 |
|  | Annual chest radiography examination for all healthcare workers | Since 2006 |
|  | Contact tracing for the neighbor patients and caregivers exposed to patients with pulmonary TB. A high-profile case of delayed respiratory isolation of a tuberculosis patient further raised physician alertness. | Since 2009 |
| Improvements in laboratory diagnosis | Bundle orders of AFS and TB culture | After 2004 |
|  | Laboratory quality assurance (mandated by Taiwan Centres for Disease Control) | Since 2004 |
|  | After clinical specimens arrive the qualified TB laboratory rooms, it is required to report the results of AFS within 24 hours with a required target of 99% or more per month |  |
|  | After clinical specimens arrive the qualified TB laboratory rooms, it is required to report the positive results of TB culture within 21 days with a required target of 60% or more per month. |  |
|  | After cultures yield *Mycobacterium* species, the result of identification is required to be reported within 7 days with a required target of 90% or more per month. |  |
|  | After *M. tuberculosis* is identified, drug susceptibility is required to be reported within 28 days with a required target of 90% or more per month. |  |
|  | Fluorescent microscopy was introduced. | Since Oct 2006 |
|  | Immediate text message to notify TB case managers and primary care physicians upon positive laboratory results of AFS, TB-PCR, and TB culture. | After Jul 2013 |
|  | The implementation of Capilia TB assay for species confirmation for the isolate by MGIT 960 (within 15 minutes). | Since 2014 |
| Expansion of isolation facilities | One intensive care unit with 14 respiratory isolation rooms specific for patients requiring respiratory isolation was built. | 2003 |
|  | Two respiratory isolation rooms in the emergency department were built. | 2004 |
|  | There were 86 respiratory isolation rooms in the hospital. | 2014 |
