# Supplementary material for: Impact of introducing fluorescent microscopy on hospital tuberculosis control: A before-after study at a high caseload medical center in Taiwan
Source: PLoS One. 2020 Apr 3;15(4):e0230067. doi: 10.1371/journal.pone.0230067 (PMC7122812; doi:10.1371/journal.pone.0230067)
Supplement: S2 Table — (DOCX) [file pone.0230067.s002.docx]

**S2 Table. Non-isolated infectious duration of hospitalized patients (at the first admission after the index culture)**

|  | 2001, median (IQR) | 2014, median (IQR) | *P* value |
| --- | --- | --- | --- |
| All tuberculosis patients |  |  |  |
| Kaplan-Meier estimate for time-to-respiratory isolation (discharge before isolation was treated as censored), days | 46 days | 19 days | 0.028 |
| Non-isolated infectious duration, median (IQR), days | 12.5 (6.8-28.3) days | 3.0 (0-8.0) days | <0.001^a^ |
| From hospital visits^b^ to the index culture date^c^, median (IQR), days | 5 (2-13) days | 2.0 (1.0-5.9) days | <0.001 |
| From sampling to the report of positive smear, mean (IQR), days | 1-2 days | 1 (1.1-1.6) days | – |
| From taking TB culture to the report of culture, mean (IQR), days | 6-8 weeks | 25.7 (18.0-29.1) days | – |
| Patient with cavitary pulmonary lesions |  |  |  |
| Non-isolated infectious duration, median (IQR), days | 3.5 (1-21) days | 0 (0-1) days | 0.001 |
| Positive sputum smear, median (IQR), days | 1 (1-8) days | 0 (0-0) days | 0.002 |
| Negative sputum smear, median (IQR), days | 9 (2-27) days | 5 (3-7) days | 0.480 |
| Patients without typical presentations |  |  |  |
| Non-isolated infectious duration, median (IQR), days | 16 (10-31.5) days | 6 (2-13) days | <0.001 |
| Positive sputum smear, median (IQR), days | 10 (7-15) days | 3 (2-8) days | 0.009 |
| Negative sputum smear, median (IQR), days | 17 (11-34.3) days | 7 (2.5-17.5) days | <0.001 |
| Patients hospitalized for comorbidities |  |  |  |
| Non-isolated infectious duration, median (IQR), days | 21 (11-45) days | 6 (2-15) days | <0.001 |
| Positive sputum smear, median (IQR), days | 10 (7.5-14) days | 3 (1-5) days | 0.015 |
| Negative sputum smear, median (IQR), days | 25 (13-46) days | 7 (5-19.5) days | <0.001 |

Data are exposure duration unless otherwise mentioned.

*P* values are based on the Mann-Whitney U-test unless specified otherwise.

AFS, acid-fast smear; IQR, interquartile range; N, patient numbers

^a^Log-rank test.

^b^Hospital visits included admission or emergent department visits.

^c^There were 11 (6.1%) patients in 2001 and 8 (9.9%) patients in 2014 who had their index cultures sent before hospital visits (P=0.307), and they were excluded in this calculation.
