# Supplementary material for: Impact of introducing fluorescent microscopy on hospital tuberculosis control: A before-after study at a high caseload medical center in Taiwan
Source: PLoS One. 2020 Apr 3;15(4):e0230067. doi: 10.1371/journal.pone.0230067 (PMC7122812; doi:10.1371/journal.pone.0230067)
Supplement: S3 Table — (DOCX) [file pone.0230067.s003.docx]

**S3 Table. Studies which investigating the factors associated with delayed respiratory isolation of hospitalized patients with pulmonary tuberculosis**

| Country [Ref] | Study population | Measurement | Results | Factors associated with delayed respiratory isolation |
| --- | --- | --- | --- | --- |
| Taiwan [PR] | 180 patients with culture-positive pulmonary TB in 2001 and 81 in 2014 | Median duration from hospital visits to respiratory isolation | 12.5 days in 2001;  3 days in 2014 | Negative sputum AFS, atypical presentations, hospitalization due to comorbidities |
| Taiwan [1] | 193 patients with culture-positive pulmonary TB | Proportion of patients with in-hospital diagnosis delay >7 days | 93 (48.2%) cases | Negative sputum smear; non-cavitary lesions on CXR, admission to non-PIW, FQ exposure, underlying malignancy, age more than 65 years |
| Taiwan [2] | 259 patients with smear/culture-  positive pulmonary TB | Proportion of patients with delayed respiratory isolation >7 days | 44 (28.3%) cases | PIW: age >70 years  Non-PIW: patients without dyspnoea, atypical CXR, admission from the ED |
| Taiwan [3] | 206 patients with smear/culture-  positive pulmonary TB | Median interval from the first medical consultation to the initiation of anti-TB treatment | 23 days | Negative sputum AFS, absence of haemoptysis, no CXR at first consultation |
| USA [4] | 765 patients with culture-positive pulmonary TB | Proportion of patients with interval between admission and ordering respiratory isolation >1 day | 172 (25.5%) cases | Absence of cough, cavitary lung lesion, and night sweats; history of non-injection drug use; receiving care at hospitals with lower rates of TB admissions |
| Canada [5] | 429 patients with culture-positive pulmonary TB | Failure to initiate respiratory isolation within the first 24 hours after admission | NA | Aged 65-79 years, born in Canada, absence of cough, concomitant extra-pulmonary disease, negative sputum AFS |

Abbreviations: AFS: acid-fast smear; CXR: chest X-ray; ED: emergency department; FQ: fluoroquinolones; ID: infectious diseases; NA: not available; PIW: pulmonary and infectious disease ward; PR: present report; Ref: references; TB: tuberculosis; USA: United State America

**References**

1. Lin CY, Lin WR, Chen TC, et al. Why is in-hospital diagnosis of pulmonary tuberculosis delayed in southern Taiwan? J Formos Med Assoc **2010**; 109(4): 269-77.

2. Hsieh MJ, Liang HW, Chiang PC, et al. Delayed suspicion, treatment and isolation of tuberculosis patients in pulmonology/infectious diseases and non-pulmonology/infectious diseases wards. J Formos Med Assoc **2009**; 108(3): 202-9.

3. Chiang CY, Chang CT, Chang RE, Li CT, Huang RM. Patient and health system delays in the diagnosis and treatment of tuberculosis in Southern Taiwan. The international journal of tuberculosis and lung disease : the official journal of the International Union against Tuberculosis and Lung Disease **2005**; 9(9): 1006-12.

4. Rozovsky-Weinberger J, Parada JP, Phan L, et al. Delays in suspicion and isolation among hospitalized persons with pulmonary tuberculosis at public and private US hospitals during 1996 to 1999. Chest **2005**; 127(1): 205-12.

5. Greenaway C, Menzies D, Fanning A, et al. Delay in diagnosis among hospitalized patients with active tuberculosis--predictors and outcomes. American journal of respiratory and critical care medicine **2002**; 165(7): 927-33.
