# Supplementary material for: Impact of introducing fluorescent microscopy on hospital tuberculosis control: A before-after study at a high caseload medical center in Taiwan
Source: PLoS One. 2020 Apr 3;15(4):e0230067. doi: 10.1371/journal.pone.0230067 (PMC7122812; doi:10.1371/journal.pone.0230067)
Supplement: S1 Fig — (DOCX) [file pone.0230067.s004.docx]

**S1 Fig. Causal mediation analyses of the effect mediated by the higher rate of early ordering (less than 4 days after admission) of smear/culture in 2014**

**
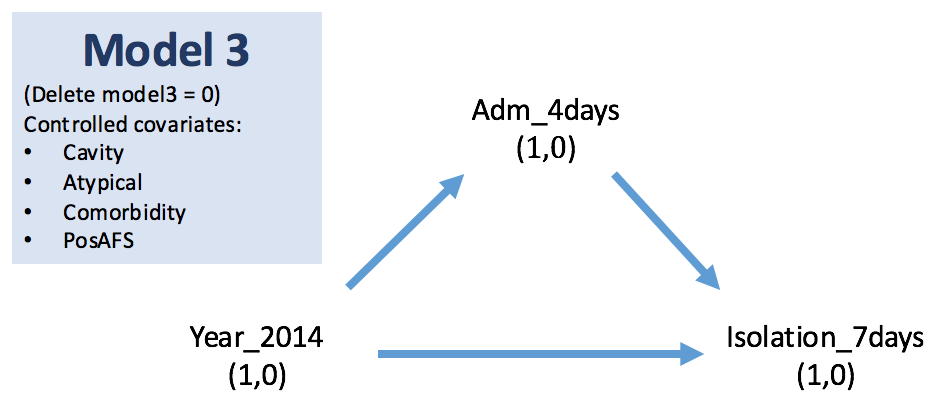
**

**
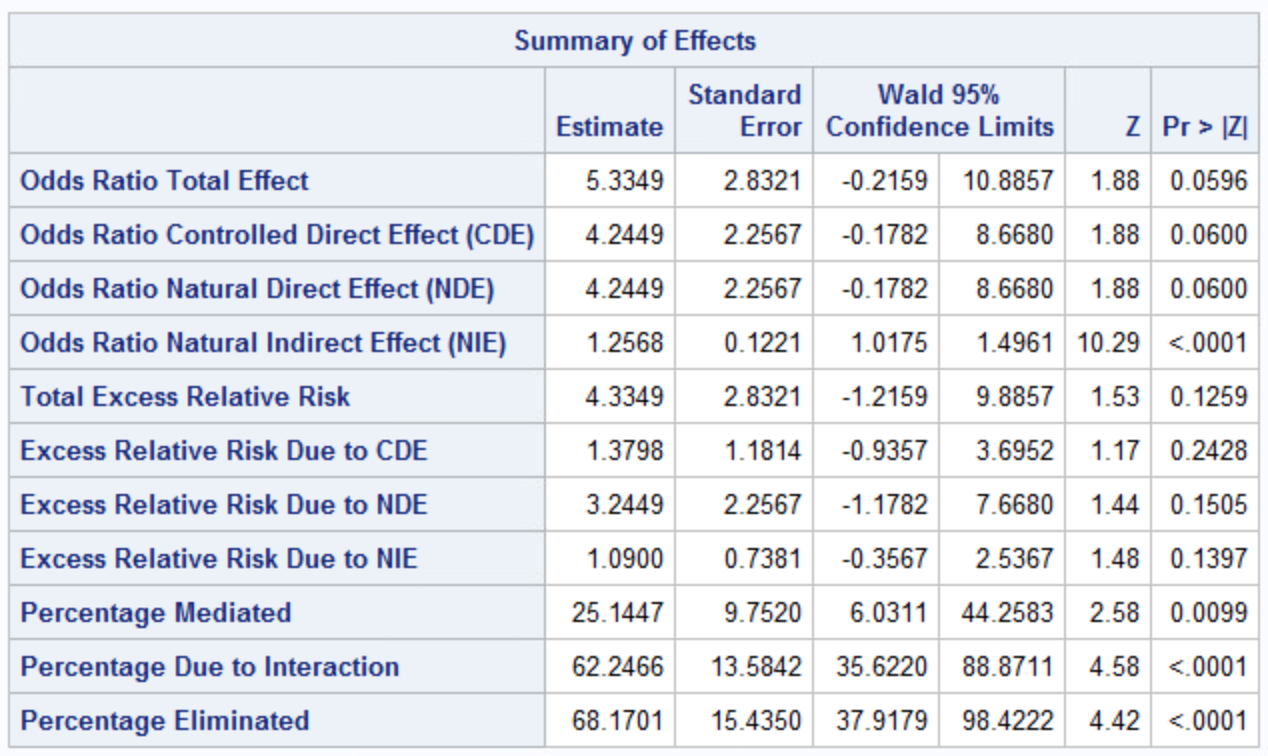
**
