# Supplementary material for: Impact of introducing fluorescent microscopy on hospital tuberculosis control: A before-after study at a high caseload medical center in Taiwan
Source: PLoS One. 2020 Apr 3;15(4):e0230067. doi: 10.1371/journal.pone.0230067 (PMC7122812; doi:10.1371/journal.pone.0230067)
Supplement: S3 Fig — (DOCX) [file pone.0230067.s006.docx]

**S3 Fig. Total non-isolated infectious patient-days in 25 medical/surgical subspecialty wards/units in 2001 and 2014**


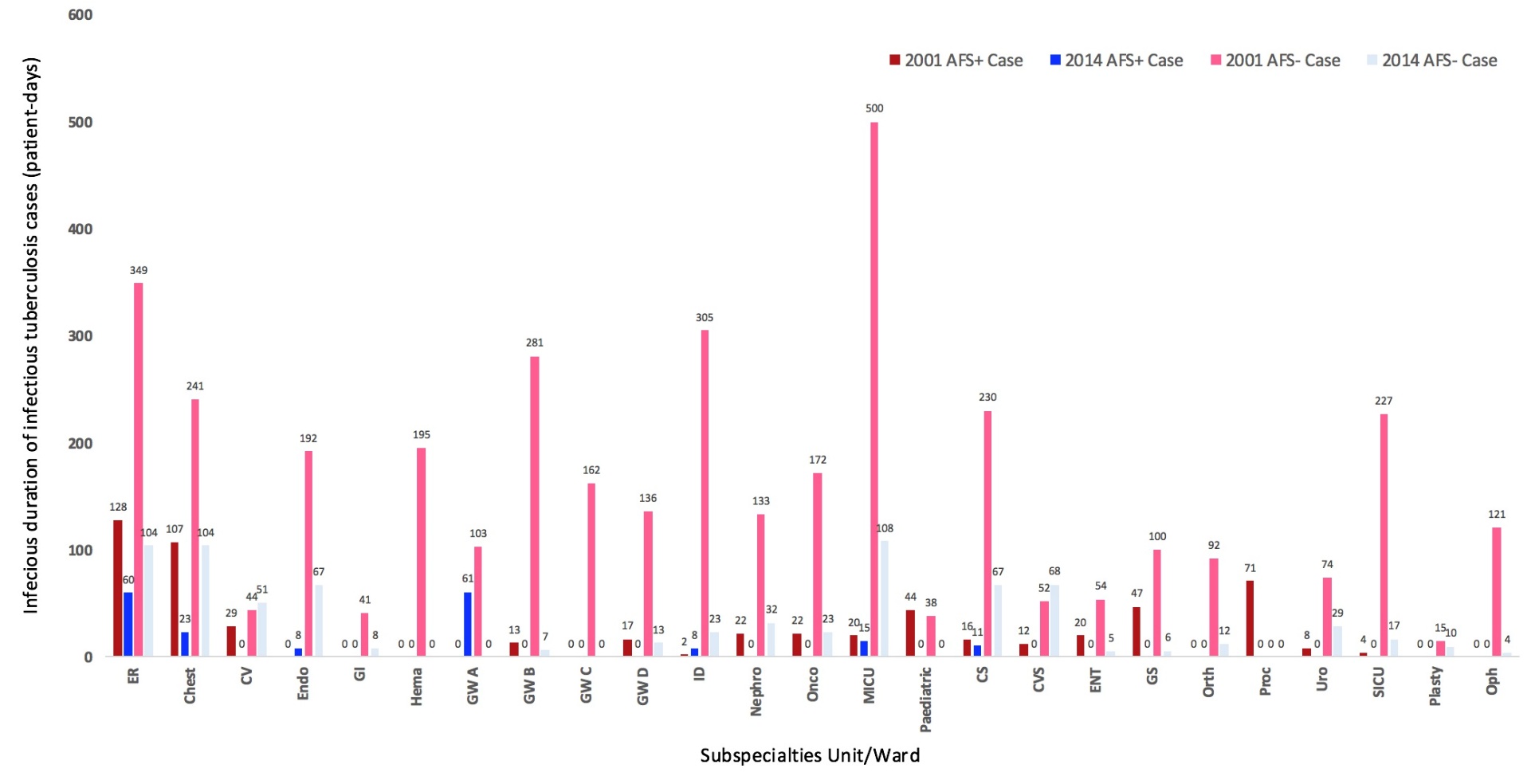


Abbreviations: CS, chest surgery; CV, cardiovascular; CVS, cardiovascular surgery; Endo, endocrinology; ENT, ear, nose, and throat; ER, emergency department; GI, gastroenterology; GS, gastrointestinal surgery; GW, general ward; Hema, haematology; ID, infectious diseases; MICU, medical intensive care unit; Nephro, nephrology; NS, neurosurgery; Onco, oncology; Oph, ophthalmology; Ortho, orthopaedics; Plasty, plastic surgery; Proc, proctology; SICU, surgical intensive care unit; Uro, urology.
